# The Efficacy and Safety of Neoadjuvant Immunotherapy in Pan-Squamous Cell Carcinomas: A Meta-Analysis of Esophageal, Head and Neck, Cervical, Lung, Cutaneous, and Oral Squamous Cell Carcinomas

**DOI:** 10.3390/jcm15156113

**Published:** 2026-08-06

**Authors:** Xiaotong Fu, Shuiqing Xu, Ming Wang

**Affiliations:** 1 Department of Gynecologic Oncology, Beijing Obstetrics and Gynecology Hospital, Capital Medical University, Beijing Maternal and Child Health Care Hospital, 17 Qihelou St., Dongcheng District, Beijing 100006, China; fuxiaotong98@163.com (X.F.); xsq@mail.ccmu.edu.cn (S.X.); 2 Department of Neurosurgery, Beijing Chao-Yang Hospital, Capital Medical University, 8 Gongren Tiyuchang Nanlu, Chaoyang District, Beijing 100191, China; 3 Department of Obstetrics and Gynecology, Beijing Chao-Yang Hospital, Capital Medical University, 8 Gongren Tiyuchang Nanlu, Chaoyang District, Beijing 100191, China; 4 Laboratory for Clinical Medicine, Capital Medical University, 10 Toutiao, You’anmenwai, Fengtai District, Beijing 100071, China

**Keywords:** neoadjuvant immunotherapy, squamous cell carcinoma, esophageal cancer, head and neck cancer, cervical cancer, lung cancer, cutaneous cancer, oral cancer, meta-analysis

## Abstract

**Background**: Squamous cell carcinomas (SCCs) share squamous differentiation and may engage the programmed cell death protein 1/programmed death-ligand 1 (PD-1/PD-L1) pathway, although their causes, biology, and treatment differ by anatomical site. We evaluated tumor response, short-term survival, and grade 3–4 adverse events after neoadjuvant immune-checkpoint blockade, with or without chemotherapy, followed by surgery. **Methods**: A comprehensive search of PubMed, Embase, the Cochrane Library, and clinical trial registries was conducted from inception to October 2024. Prospective and retrospective studies evaluating neoadjuvant immunotherapy, with or without chemotherapy, before surgery in patients with SCC were included. In mixed-histology studies, data were eligible only when SCC outcomes could be extracted separately. Pooled proportions and 95% confidence intervals (CIs) were estimated with random-effects models. **Results**: A total of 44 studies involving 2586 patients with six anatomical SCC groups were included. The pooled objective response rate (ORR) was 0.70 (95% CI, 0.59–0.80), clinical complete response (cCR) rate was 0.17 (95% CI, 0.10–0.28), and pathological complete response (pCR) rate was 0.30 (95% CI, 0.27–0.34). The pooled 1-year progression-free survival (PFS), disease-free survival (DFS), and overall survival (OS) rates were 0.82 (95% CI, 0.79–0.86), 0.86 (95% CI, 0.75–0.92), and 0.92 (95% CI, 0.90–0.93), respectively. The pooled incidence of grade 3–4 adverse events was 0.18 (95% CI, 0.14–0.23). Because the PD-L1 subgroup contained only one study for most outcomes, checkpoint-target subgroup findings were considered exploratory. **Conclusions**: Existing predominantly single-arm evidence suggests antitumor activity of neoadjuvant immune-checkpoint blockade, with or without chemotherapy, in selected patients with resectable SCC. Because esophageal SCC accounted for most studies and clinical heterogeneity was substantial, these pooled proportions should not be interpreted as comparative effects or as evidence of uniform benefit across SCC sites.

## 1. Introduction

Squamous cell carcinomas (SCCs) arise at diverse anatomical sites but share squamous differentiation. In 2024, cancers of the esophagus, oral cavity and pharynx, respiratory system, and uterine cervix collectively accounted for a substantial cancer burden in the United States [1]. Neoadjuvant chemotherapy can reduce tumor burden before surgery, but pathological complete response rates with chemotherapy alone remain modest in several settings. Reported pCR rates are approximately 11.5% in locally advanced cervical cancer, 2.2% in resectable non-small-cell lung cancer, and 9% in esophageal SCC [2,3,4]. In oral cavity SCC, long-term follow-up of a randomized trial found no survival benefit from preoperative chemotherapy [5]. For resectable cutaneous SCC, surgery remains the principal curative treatment, whereas locally advanced disease may require multimodal management [6]. These limitations have prompted investigation of more effective neoadjuvant systemic strategies.

The causes and molecular features of SCC vary substantially by anatomical site. High-risk human papillomavirus (HPV) accounts for a large share of cervical and oropharyngeal cancers, whereas the HPV-attributable fraction is substantially lower in cancers of the oral cavity [7]. Ultraviolet radiation is a major cause of cutaneous SCC, whereas tobacco, alcohol, and other exposures contribute to SCCs arising in the aerodigestive tract. Despite this heterogeneity, many SCCs develop immune-evasion programs within the tumor microenvironment, including engagement of the PD-1/PD-L1 pathway. This shared, although not uniform, immunobiology provides a rationale for examining immune-checkpoint blockade across anatomical SCC sites [8,9].

Immune-checkpoint inhibitors restore antitumor T-cell activity principally by blocking the PD-1/PD-L1 pathway [8,9]. CTLA-4 blockade was represented only in a limited combination setting in the included evidence and was therefore not treated as a principal intervention class in this review. Chemotherapy may complement checkpoint blockade through tumor-cell killing, antigen release, and immunogenic cell death, providing a biological rationale for neoadjuvant chemoimmunotherapy [10]. Clinical evidence has consequently expanded across several SCC settings, including esophageal SCC, head and neck SCC, oral cavity SCC, lung SCC, and cutaneous SCC [3,11,12,13,14,15]. However, activity estimates vary across tumor sites, regimens, and outcome definitions, and evidence outside esophageal SCC remains comparatively limited.

In this review, “pan-SCC” is used only as an evidence-synthesis framework for SCCs from several anatomical sites; it does not imply a single disease entity or a uniform treatment effect. Efficacy outcomes were assessed through radiological response, pathological response, and the specific 1-year survival outcomes of progression-free survival (PFS), disease-free survival (DFS), and overall survival (OS). Safety was summarized using the grade 3–4 adverse-event endpoint reported by each study. Because attribution and observation windows were not uniform, this pooled estimate was interpreted descriptively rather than as a common treatment-related toxicity definition. We therefore synthesized neoadjuvant immune-checkpoint blockade given alone or with chemotherapy before surgery and examined anatomical-site and checkpoint-target subgroups cautiously.

## 2. Materials and Methods

### 2.1. Search Strategy and Selection Criteria

This systematic review and meta-analysis was conducted in accordance with the Preferred Reporting Items for Systematic Reviews and Meta-Analyses (PRISMA) 2020 statement [16]. The protocol was registered in PROSPERO (CRD420261342127). PubMed, Embase, the Cochrane Library, ClinicalTrials.gov, and the WHO International Clinical Trials Registry Platform were searched from inception to October 2024. Search terms covered neoadjuvant immunotherapy, immune-checkpoint inhibitors, individual agents, and SCCs of the esophagus, head and neck, cervix, lung, skin, and oral cavity. Reference lists of relevant articles and reviews were also screened. Complete database- and registry-specific search strategies are provided in Supplementary Table S7. PRISMA checklist is provided in Supplementary Table S8.

Studies were included if they met the following criteria: (1) prospective or retrospective clinical studies, including single-arm trials and observational cohorts; (2) patients received neoadjuvant immunotherapy, with or without chemotherapy, in a preoperative treatment pathway; (3) patients had SCC of the esophagus, head and neck, cervix, lung, skin, or oral cavity; and (4) treatment response or survival outcomes were reported. Conversion-to-surgery studies were eligible only when systemic treatment was delivered with prospectively stated curative-intent resection as the planned endpoint, and the surgical cohort or intention-to-treat population could be identified; studies in which surgery was an unplanned option after treatment for initially palliative or unresectable disease were excluded. Studies enrolling mixed histological populations were eligible only when SCC subgroup outcomes could be extracted separately. Exclusion criteria were case reports, conference abstracts, animal studies, non-neoadjuvant or non-surgical settings, duplicate or overlapping cohorts, and studies without separately extractable SCC outcomes.

After duplicate removal, two reviewers (FXT and XSQ) independently screened titles and abstracts and then assessed potentially eligible full texts. Disagreements were resolved by discussion or consultation with a third reviewer (MW). For multiple reports from the same cohort, the most complete report was used for each outcome and overlapping participants were not counted twice. Mixed-histology studies were retained only when SCC data were separately extractable; a study could contribute to the descriptive characteristics without contributing to an endpoint meta-analysis when SCC-specific events were unavailable. The studies contributing to each endpoint are listed in Supplementary Table S2.

### 2.2. Data Extraction and Quality Assessment

Two reviewers independently extracted study characteristics, patient and tumor features, intervention details, and outcome data using a standardized form. Disagreements were resolved by consensus. For each endpoint, event counts and analysis denominators were used when available. When a report or the original analysis file provided only a study-level proportion and its 95% CI, that estimate and CI were retained without reconstructing unreported integer counts; its logit standard error was derived from the CI.

Methodological quality was assessed using the validated Methodological Index for Non-Randomized Studies (MINORS). The eight-item version was applied to non-comparative studies, whereas the four additional comparative domains were evaluated for studies containing a relevant comparison group. Each item was scored 0 (not reported), 1 (reported but inadequate), or 2 (reported and adequate). Judgments followed the original MINORS definitions, with follow-up evaluated against the main endpoint and documented treatment-pathway attrition distinguished from loss to follow-up. Study-level domain scores are presented in Supplementary Table S6. No post hoc low-, moderate-, or high-quality cutoffs were used because these are not part of the validated instrument.

Primary efficacy outcomes were objective response rate (ORR), clinical complete response (cCR), and pathological complete response (pCR). ORR was the proportion achieving complete or partial radiological response according to the criteria used in each study. cCR denoted complete clinical or radiological response and was not treated as interchangeable with pathological complete response. pCR denoted no viable tumor in the resection specimen, according to the included study definition. Secondary efficacy outcomes were partial response (PR), major pathological response (MPR), and 1-year progression-free survival (PFS), disease-free survival (DFS), and overall survival (OS); the safety outcome was the incidence of study-reported grade 3–4 adverse events. PR generally followed RECIST 1.1 when reported [17], whereas MPR was generally defined as no more than 10% viable tumor. For survival outcomes, a directly reported 12-month estimate was preferred. When this was unavailable, the estimate at the nearest reported time point was used only if it fell within 9–15 months; otherwise, the study did not contribute to that 1-year outcome. When necessary, 12-month survival was read from the published Kaplan–Meier curve. Endpoint availability, the analysis population, extraction source, and study-specific definitions followed each report and are documented in Supplementary Table S2.

### 2.3. Statistical Analysis

Meta-analyses were performed using RevMan (version 5.4) and R software (version 4.3.0). Study-specific proportions were transformed to the logit scale and pooled using a general inverse-variance random-effects model with the DerSimonian–Laird estimator [18]; summary estimates and 95% confidence intervals (CIs) were subsequently back-transformed to the proportion scale. For studies with zero events or events in all participants, a 0.5 continuity correction was applied. When an event count and denominator were reported, these values were used directly. When only a proportion and its 95% CI were available, the corresponding logit-transformed estimate and sampling variance were reconstructed from the reported interval before pooling. The same proportion-based framework was used for 1-year PFS, DFS, and OS. Heterogeneity was quantified using the I^2^ statistic, with I^2^ > 50% considered substantial. Random-effects models were used throughout because clinical and methodological heterogeneity was anticipated. Subgroup analyses were conducted according to anatomical site and checkpoint target, including PD-1-versus PD-L1-based treatment. Leave-one-out sensitivity analyses evaluated the robustness of the pooled estimates. Funnel plots and Egger’s regression test were used to explore small-study effects when at least 10 studies were available; funnel-plot asymmetry was not interpreted as proof of publication bias. All tests were two-sided, and *p* < 0.05 was considered statistically significant.

## 3. Results

### 3.1. Study Selection and Characteristics

**Figure 1:**
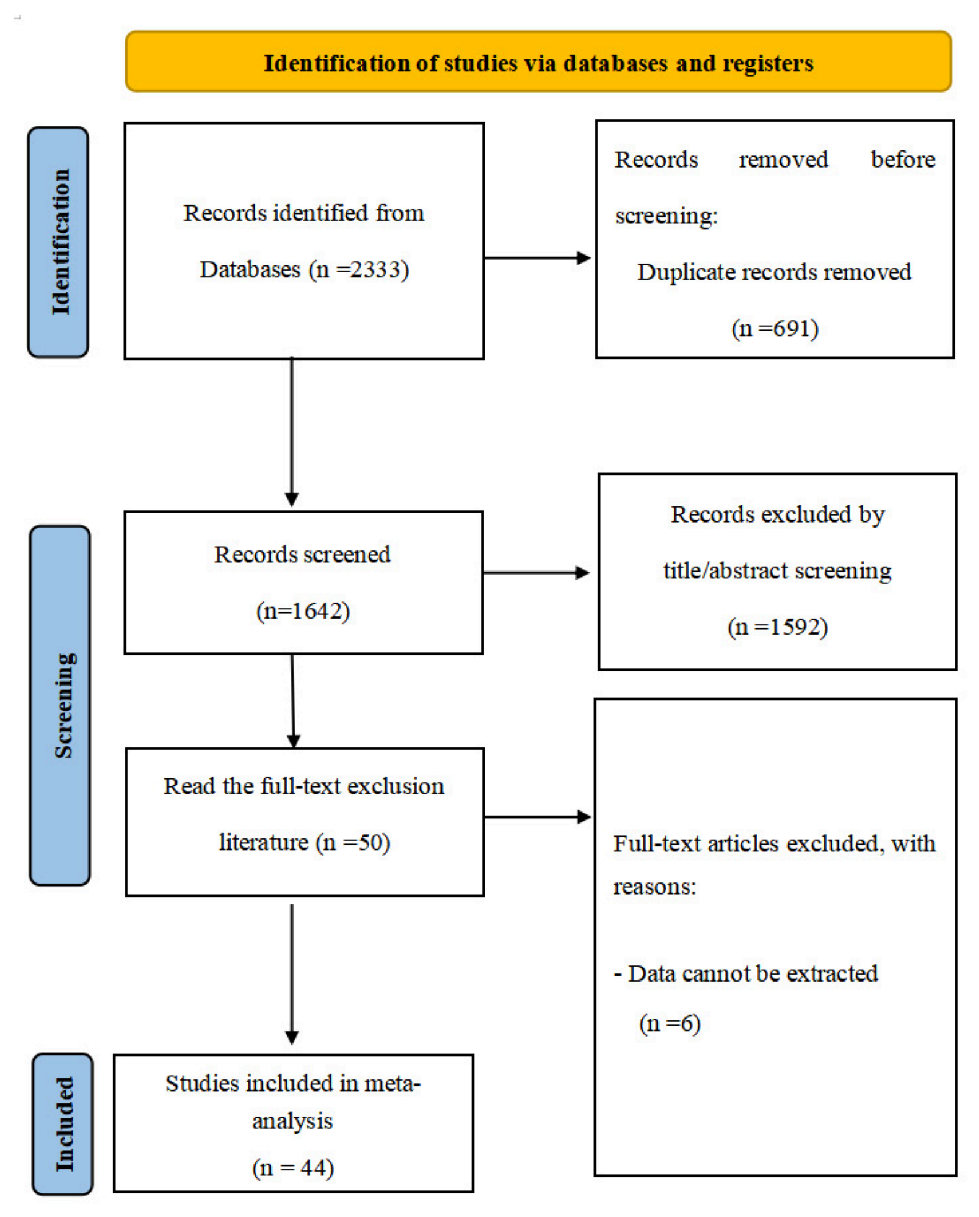
Flow chart of literature screening.

The initial search yielded 2333 records. After screening and histology-specific re-evaluation, 44 studies involving 2586 patients met the eligibility criteria and were included (Figure 1). Three mixed-histology NSCLC studies were excluded because SCC-specific outcomes could not be extracted separately [19,20,21]. The evidence base consisted predominantly of prospective single-arm clinical studies and retrospective observational studies; no randomized controlled trials were identified. The 44 included reports are detailed in Supplementary Table S1 [22,23,24,25,26,27,28,29,30,31,32,33,34,35,36,37,38,39,40,41,42,43,44,45,46,47,48,49,50,51,52,53,54,55,56,57,58,59,60,61,62,63,64,65].

The tumor types were esophageal SCC (29 studies), head and neck SCC (8), lung SCC (3), oral SCC (2), cervical SCC (1), and cutaneous SCC (1). No eligible basket trial contributed the same participants to more than one anatomical category. In mixed-histology studies, only separately extractable SCC data were used; Guangyin Zhao 2023 [51] contributed its 16-patient SCC subgroup to descriptive characteristics only because SCC-specific endpoint events could not be separated. The MINORS assessment identified recurrent limitations across the evidence base (Supplementary Table S6).

### 3.2. Treatment Response of Neoadjuvant Immune Checkpoint Inhibitors to SCC

**Figure 2:**
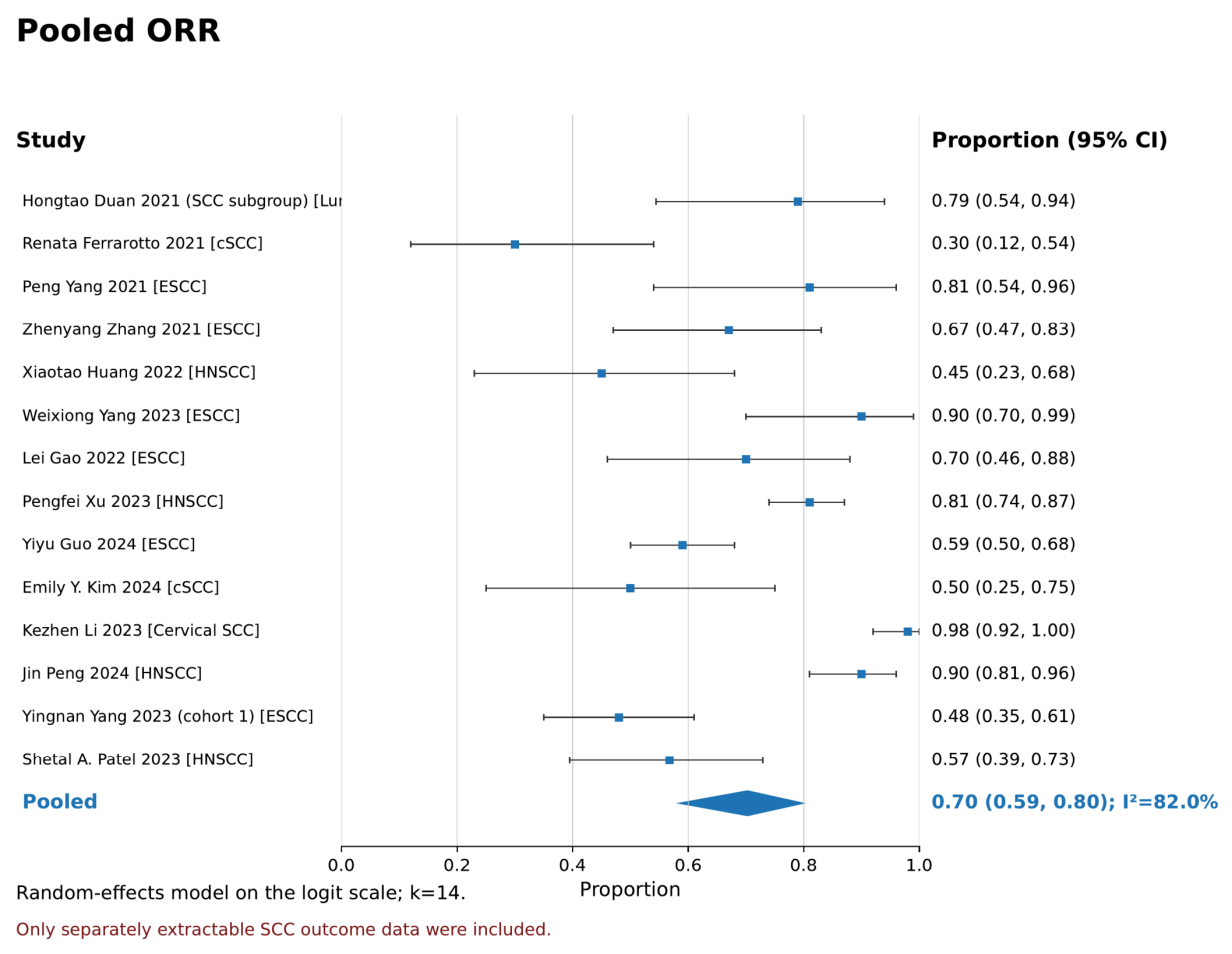
Forest plot of the objective response rate (ORR) following neoadjuvant immunotherapy-based treatment across squamous cell carcinoma sites. The pooled ORR was 0.70 (95% CI, 0.59–0.80; I^2^ = 82.0%). Only separately extractable SCC outcome data were included. Squares represent the proportion estimate of individual studies. Horizontal lines denote 95% confidence intervals. The diamond indicates the pooled proportion and its 95% confidence interval.

In total, 14 studies were included in the ORR analysis. The pooled ORR was 0.70 (95% CI, 0.59–0.80; I^2^ = 82.0%) using a random-effects model (Figure 2).

**Figure 3:**
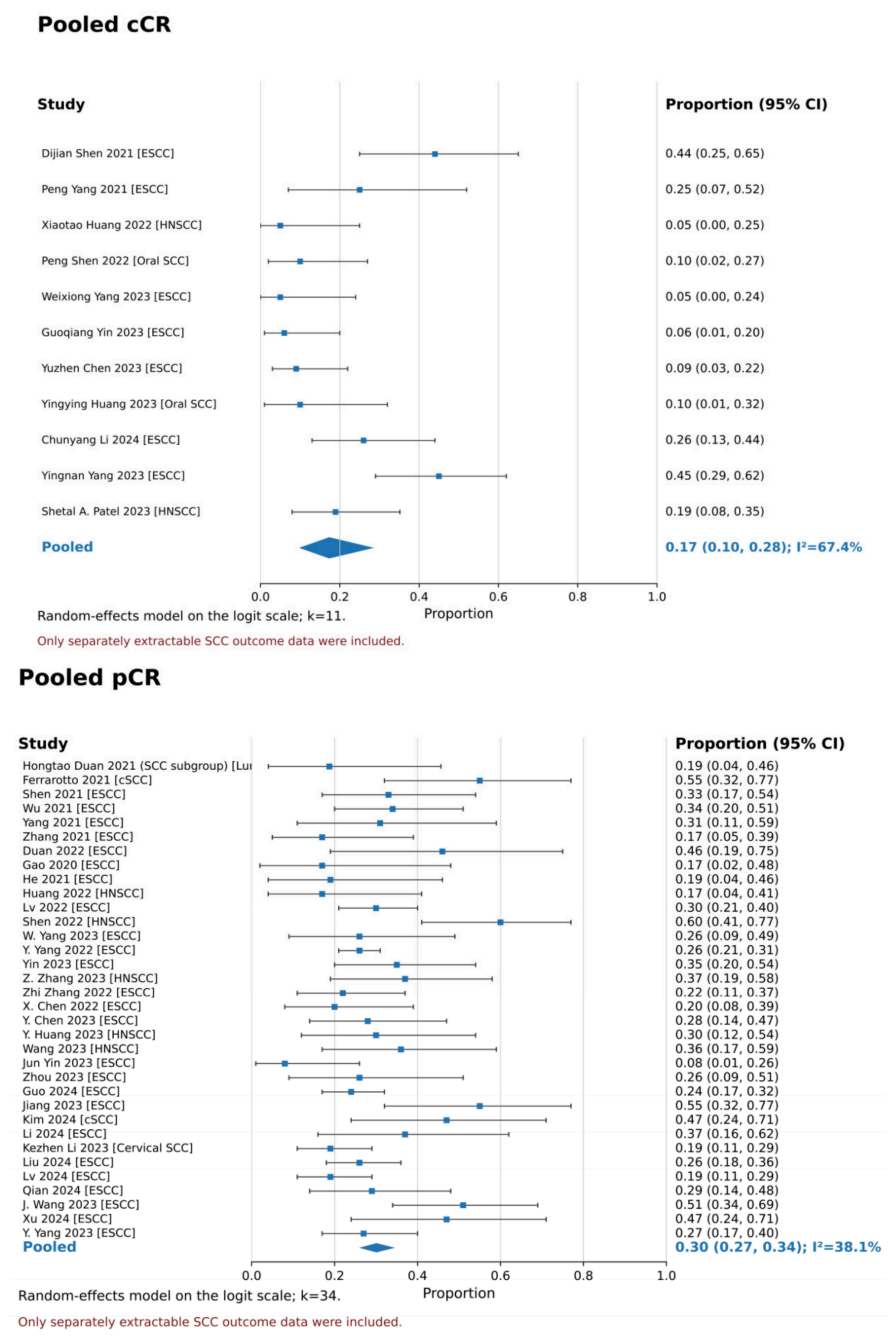
Forest plots of complete response outcomes following neoadjuvant immunotherapy-based treatment. The upper panel shows the clinical complete response (cCR), with a pooled rate of 0.17 (95% CI, 0.10–0.28; I^2^ = 67.4%), and the lower panel shows the pathological complete response (pCR), with a pooled rate of 0.30 (95% CI, 0.27–0.34; I^2^ = 38.1%). Only separately extractable SCC outcome data were included. Squares denote the proportion of each individual study; horizontal lines represent 95% confidence intervals; the diamond indicates the pooled proportion and its corresponding 95% confidence interval.

A total of 11 studies contributed to the cCR analysis, yielding a pooled rate of 0.17 (95% CI, 0.10–0.28; I^2^ = 67.4%). Thirty-four studies contributed to the pCR analysis, with a pooled rate of 0.30 (95% CI, 0.27–0.34; I^2^ = 38.1%) (Figure 3).

**Figure 4:**
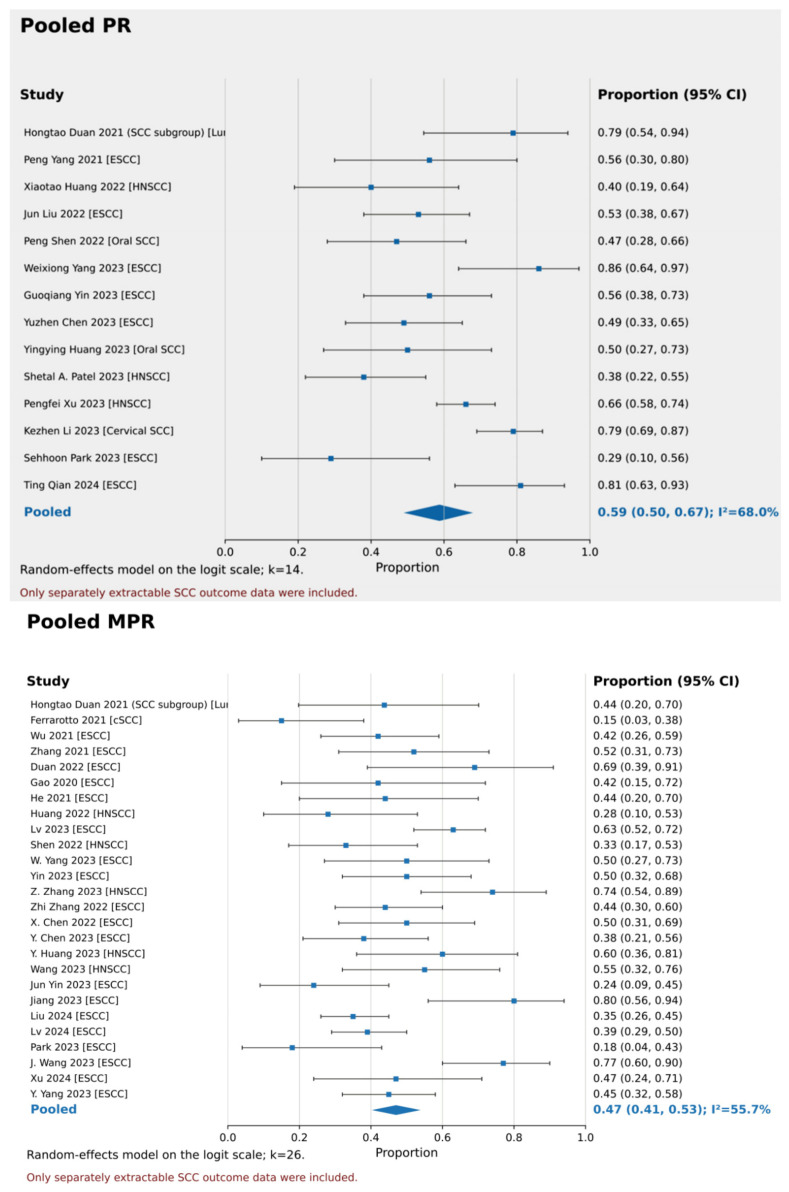
Forest plots of radiological and pathological response outcomes following neoadjuvant immunotherapy-based treatment. The upper panel shows the partial response (PR), with a pooled rate of 0.59 (95% CI, 0.50–0.67; I^2^ = 68.0%), and the lower panel shows the major pathological response (MPR), with a pooled rate of 0.47 (95% CI, 0.41–0.53; I^2^ = 55.7%). Only separately extractable SCC outcome data were included. Squares denote the proportion of each individual study; horizontal lines represent 95% confidence intervals; the diamond indicates the pooled proportion and its corresponding 95% confidence interval.

In total, 14 studies were included in the PR analysis, with a pooled rate of 0.59 (95% CI, 0.50–0.67; I^2^ = 68.0%). Twenty-six studies contributed to the MPR analysis, yielding a pooled rate of 0.47 (95% CI, 0.41–0.53; I^2^ = 55.7%) (Figure 4).

### 3.3. Survival and Safety Outcomes

**Figure 5:**
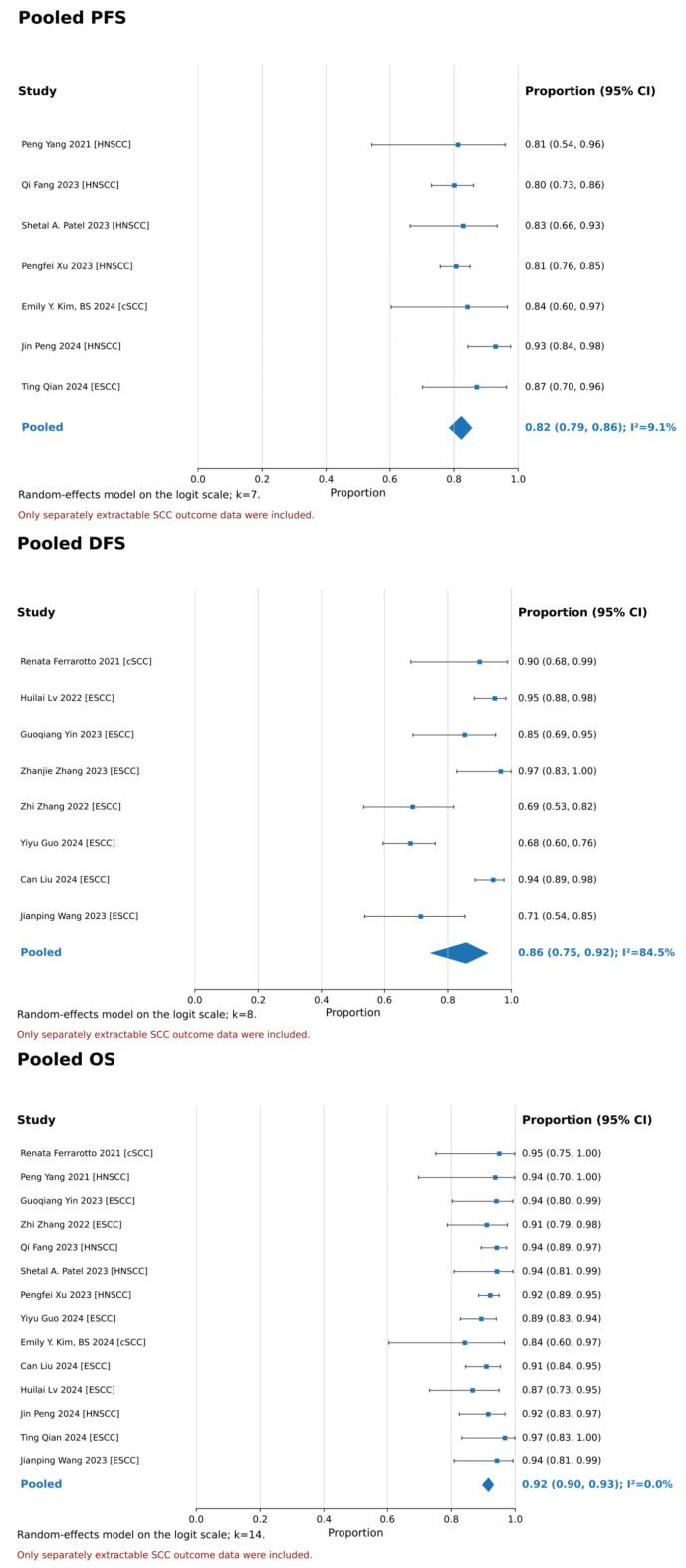
Forest plots of 1-year survival outcomes following neoadjuvant immunotherapy-based treatment. The upper panel shows 1-year progression-free survival (PFS), with a pooled rate of 0.82 (95% CI, 0.79–0.86; I^2^ = 9.1%); the middle panel shows 1-year disease-free survival (DFS), with a pooled rate of 0.86 (95% CI, 0.75–0.92; I^2^ = 84.5%); and the lower panel shows 1-year overall survival (OS), with a pooled rate of 0.92 (95% CI, 0.90–0.93; I^2^ = 0.0%). Only separately extractable SCC outcome data were included. Squares denote the proportion of each individual study; horizontal lines represent 95% confidence intervals; the diamond indicates the pooled proportion and its corresponding 95% confidence interval.

Seven studies were included in the 1-year PFS analysis, yielding a pooled rate of 0.82 (95% CI, 0.79–0.86; I^2^ = 9.1%). Eight studies contributed to the 1-year DFS analysis, with a pooled rate of 0.86 (95% CI, 0.75–0.92; I^2^ = 84.5%). Fourteen studies contributed to the 1-year OS analysis, with a pooled rate of 0.92 (95% CI, 0.90–0.93; I^2^ = 0.0%) (Figure 5).

A total of 20 studies were included in the safety analysis. The pooled incidence of grade 3–4 adverse events was 0.18 (95% CI, 0.14–0.23; I^2^ = 67.8%) (Supplementary Figure S1).

Anatomical-site subgroup estimates (esophageal vs. non-esophageal SCC) were as follows: ORR: 0.63 (0.52–0.73; k = 6) vs. 0.72 (0.53–0.86; k = 8), *p* for subgroup = 0.386; cCR: 0.21 (0.11–0.37; k = 7) vs. 0.13 (0.07–0.23; k = 4), *p* for subgroup = 0.281; pCR: 0.28 (0.25–0.32; k = 25) vs. 0.35 (0.24–0.47; k = 9), *p* for subgroup = 0.274; PR: 0.58 (0.45–0.70; k = 7) vs. 0.59 (0.45–0.71; k = 7), *p* for subgroup = 0.960; MPR: 0.47 (0.41–0.54; k = 19) vs. 0.45 (0.30–0.60; k = 7), *p* for subgroup = 0.770; PFS: 0.85 (0.72–0.93; k = 2) vs. 0.83 (0.78–0.87; k = 5), *p* for subgroup = 0.714; DFS: 0.83 (0.70–0.92; k = 6) vs. 0.93 (0.81–0.98; k = 2), *p* for subgroup = 0.166; OS: 0.91 (0.88–0.93; k = 8) vs. 0.92 (0.90–0.94; k = 6), *p* for subgroup = 0.321; AE: 0.20 (0.15–0.26; k = 15) vs. 0.11 (0.07–0.19; k = 5), *p* for subgroup = 0.063. These observational subgroup comparisons were exploratory and were not adjusted for regimen, study design, or patient mix. Leave-one-out analyses did not reverse the direction of any pooled estimate; complete results are provided in Supplementary Table S3. PD-1/PD-L1 analyses remained exploratory because the PD-L1 subgroup generally contained one study. Exploratory Egger tests suggested funnel asymmetry for pCR, grade 3–4 adverse events, and OS (all *p* ≤ 0.001), but not for MPR, ORR, cCR, or PR. Given the single-arm proportion design and repeated or highly similar estimates, these findings were not interpreted as proof of publication bias (Supplementary Table S5).

**Figure 6:**
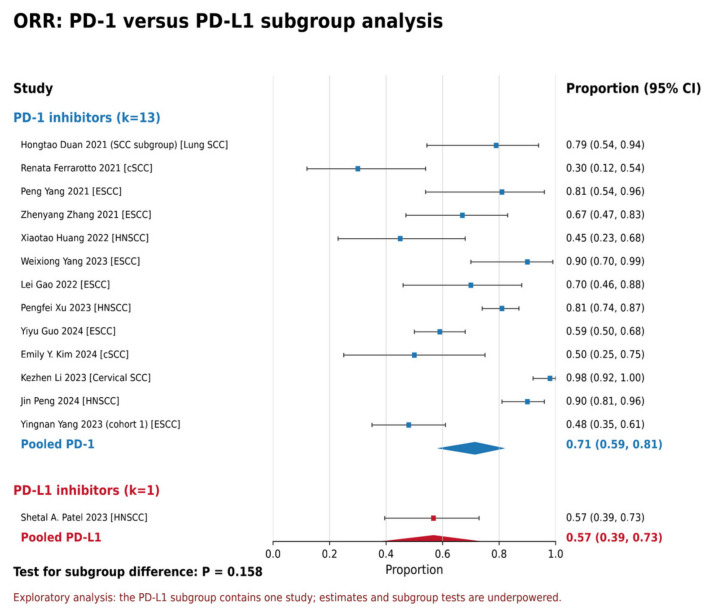

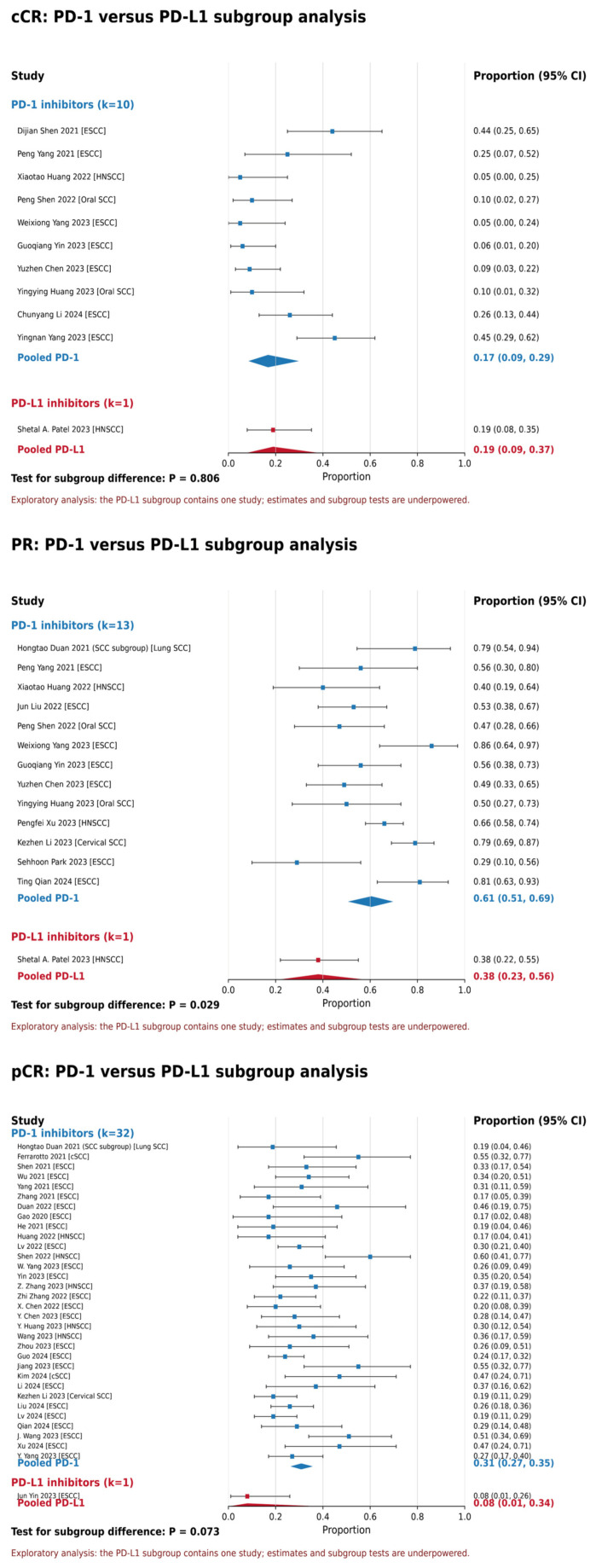

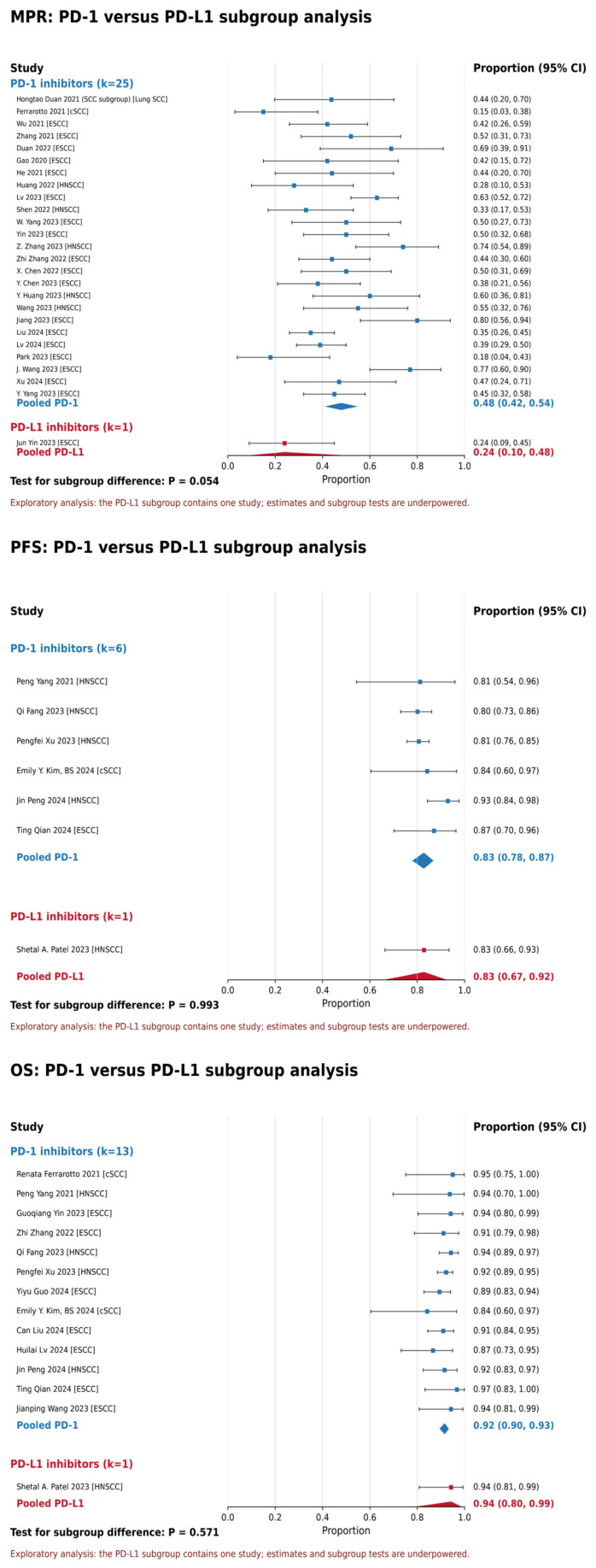
Exploratory subgroup analyses of PD-1- versus PD-L1-based neoadjuvant treatment. Forest plots are presented for objective response rate (ORR), clinical complete response (cCR), partial response (PR), pathological complete response (pCR), major pathological response (MPR), 1-year progression-free survival (PFS), and 1-year overall survival (OS). The pooled PD-1 and PD-L1 estimates were 0.71 (95% CI, 0.59–0.81; I^2^ = 83.0%) and 0.57 (95% CI, 0.39–0.73; I^2^ = 0%) for ORR; 0.17 (95% CI, 0.09–0.29; I^2^ = 70.3%) and 0.19 (95% CI, 0.09–0.37; I^2^ = 0%) for cCR; 0.61 (95% CI, 0.51–0.69; I^2^ = 64.8%) and 0.38 (95% CI, 0.23–0.56; I^2^ = 0%) for PR; 0.31 (95% CI, 0.27–0.35; I^2^ = 36.5%) and 0.08 (95% CI, 0.01–0.34; I^2^ = 0%) for pCR; 0.48 (95% CI, 0.42–0.54; I^2^ = 54.5%) and 0.24 (95% CI, 0.10–0.48; I^2^ = 0%) for MPR; 0.83 (95% CI, 0.78–0.87; I^2^ = 24.1%) and 0.83 (95% CI, 0.67–0.92; I^2^ = 0%) for 1-year PFS; and 0.92 (95% CI, 0.90–0.93; I^2^ = 0%) and 0.94 (95% CI, 0.80–0.99; I^2^ = 0%) for 1-year OS, respectively. Because the PD-L1 subgroup contained only one study for each displayed outcome, these findings are descriptive and do not support definitive comparisons between PD-1- and PD-L1-based treatment. Blue squares and diamond correspond to individual studies and pooled estimate for the PD-1 inhibitor subgroup. Red squares and diamond correspond to individual studies and pooled estimate for the PD-L1 inhibitor subgroup. Squares denote the proportion of each individual study; horizontal lines represent 95% confidence intervals; the diamond indicates the pooled proportion and its corresponding 95% confidence interval.

Exploratory subgroup analyses showed pooled rates of 0.71 versus 0.57 for ORR, 0.17 versus 0.19 for cCR, 0.61 versus 0.38 for PR, 0.31 versus 0.08 for pCR, 0.48 versus 0.24 for MPR, 0.83 versus 0.83 for 1-year PFS, and 0.92 versus 0.94 for 1-year OS in the PD-1 and PD-L1 subgroups, respectively (Figure 6). However, the PD-L1 subgroup contained only one study for each outcome; therefore, these numerical differences were exploratory and should not be interpreted as comparative treatment effects (Figure 6).

## 4. Discussion

### 4.1. Principal Findings

This review synthesized 44 predominantly single-arm or observational studies involving 2586 patients. Pooled estimates suggested radiological and pathological activity, but heterogeneity was moderate to substantial for several endpoints. These proportions describe outcomes in selected study cohorts and do not establish superiority over chemotherapy, surgery-first strategies, or other controls.

### 4.2. Site-Specific Interpretation

Most included studies evaluated esophageal SCC. A 2024 systematic review of neoadjuvant immunotherapy plus chemotherapy for resectable esophageal cancer also reported substantial pathological activity, although heterogeneity across regimens and predominantly nonrandomized evidence limited causal conclusions [11]. Individual clinical studies have likewise reported encouraging pathological responses after neoadjuvant chemoimmunotherapy in resectable esophageal SCC [19]. These findings are broadly consistent with the pooled response estimates in the present analysis, but they do not establish superiority over chemotherapy alone.

Evidence from non-esophageal SCCs is less mature and should be interpreted by anatomical site. In resectable head and neck SCC, phase 2 studies of perioperative pembrolizumab demonstrated feasibility and pathological tumor responses [12,13]. In untreated oral cavity SCC, a randomized phase 2 trial of neoadjuvant nivolumab with or without ipilimumab showed pathological responses without delaying surgery [14]. In resectable stage II–IV cutaneous SCC, neoadjuvant cemiplimab produced a centrally reviewed pCR in 51% of patients and an MPR in 13% [15]; follow-up suggested that pathological response was associated with favorable early outcomes [66]. These site-specific studies support continued investigation but do not justify assuming a uniform treatment effect across all SCCs.

### 4.3. Clinical Implications

Treatment response to immune-checkpoint blockade may vary with tumor type, immune contexture, and treatment regimen. Biomarkers were not analyzed consistently across the included studies, and this meta-analysis was not designed to estimate biomarker-specific effects. Consequently, the pooled estimates and cross-site numerical differences should not be interpreted as biomarker-adjusted treatment effects. The exploratory PD-1 versus PD-L1 analysis is especially limited because the PD-L1 subgroup contained only one study; the observed PR difference therefore does not establish greater efficacy of PD-1 inhibitors.

The pooled safety estimate also requires cautious interpretation because included studies did not always use identical definitions for treatment-related, immune-related, and all-cause adverse events. In the phase 2 cutaneous SCC study, grade 3 or higher adverse events during the study period occurred in 18% of patients [15]. Across tumor sites, early recognition and organ-specific management of immune-related toxicity remain essential, but direct comparison of adverse-event rates is appropriate only when event definitions and observation periods are aligned.

### 4.4. Strengths and Limitations

A strength of this review is its broad, anatomically explicit synthesis of radiological response, pathological response, short-term survival, and safety outcomes within one predefined framework. Mapping each study’s contribution by endpoint and treating checkpoint-target analyses as exploratory make the boundaries of the evidence more transparent. The esophageal versus non-esophageal analysis was included as an exploratory description of the dominant evidence base, not as a methodological strength or as a biologically homogeneous comparison; the non-esophageal category combines distinct diseases. Clinically, the findings are best used to inform trial design and multidisciplinary discussion rather than to select a uniform neoadjuvant regimen across SCC sites.

The present analysis has several limitations. First, it is a single-arm pooled-proportion meta-analysis without a direct comparator; therefore, the estimates cannot demonstrate superiority over neoadjuvant chemotherapy or other standards. Second, substantial clinical heterogeneity exists across anatomical sites, etiologies, immune microenvironments, regimens, analytic populations, and follow-up schedules. Definitions and ascertainment of cCR, pCR, MPR, and grade 3–4 safety outcomes also varied among studies and probably contributed to the observed heterogeneity. Third, the evidence base was dominated by esophageal SCC, whereas several other sites were represented by few studies and the combined non-esophageal category remained biologically heterogeneous. Fourth, follow-up was generally short, limiting interpretation of long-term survival. Fifth, the MINORS assessment showed recurrent weaknesses in consecutive enrollment, independent assessment of the main endpoint, prospective sample-size calculation, and, among comparative studies, control-group comparability and confounding adjustment. These limitations, together with the predominance of uncontrolled single-arm studies, reduce confidence in the precision and generalizability of the pooled proportions. Finally, the exploratory PD-1/PD-L1 comparison was markedly unbalanced and may reflect study-level confounding rather than a target-specific effect. Future randomized trials and site-specific analyses should use standardized response and safety definitions and prospectively specified, independently assessed endpoints.

## 5. Conclusions

In summary, current evidence supports continued evaluation of neoadjuvant immune-checkpoint blockade in resectable SCC, particularly in site-specific randomized trials. The predominance of esophageal SCC, sparse evidence for several other sites, variable regimens and endpoint definitions, and the single-arm nature of most studies preclude a uniform pan-SCC treatment conclusion.

## Data Availability

The datasets supporting the conclusions of this article are included within the manuscript and its Supplementary Materials. Further raw extracted datasets are available upon reasonable request from the corresponding author.

## Supplementary Materials

The following supporting information can be downloaded at: https://www.mdpi.com/article/10.3390/jcm15156113/s1.

## Abbreviations

The following abbreviations are used in this manuscript:

ACSCC	Anal Canal Squamous Cell Carcinoma
CI	Confidence Interval
CPS	Combined Positive Score
CR	Complete Response
DFS	Disease-Free Survival
EMT	Epithelial–Mesenchymal Transition
ICI	Immune Checkpoint Inhibitor
irAE	Immune-Related Adverse Event
PFS	Progression-Free Survival
OS	Overall Survival
TMB	Tumor Mutation Burden
TME	Tumor Microenvironment
