# Supplementary material for: The Efficacy and Safety of Neoadjuvant Immunotherapy in Pan-Squamous Cell Carcinomas: A Meta-Analysis of Esophageal, Head and Neck, Cervical, Lung, Cutaneous, and Oral Squamous Cell Carcinomas
Source: J Clin Med. 2026 Aug 6;15(15):6113. doi: 10.3390/jcm15156113 (PMC13466374; doi:10.3390/jcm15156113)
Supplement: Supplementary file 1 [file jcm-15-06113-s001.zip › Supplementary Table S1.pdf]

Supplementary Table S1. Characteristics of the 44 included studies.

NO.	Author	Year	Country	Study Type	Number	Age	cancer sites	Intervention
1	Hongtao Duan	2021	China	single-arm observational study	19 (SCC subgroup)	61.83	Lung squamous cell carcinoma subgroup	conventional chemotherapy regimen with PD-1 inhibitors
2	Renata Ferrarotto	2021	USA	single-institution, pilot phase II study	20	68.4 (10.9)	Head and neck squamous cell carcinoma (HNSCC)	cemiplimab and adjuvant radiotherapy or chemoradiation
3	Dijian Shen	2021	China	single-group study	28	62.2 (48–79)	Esophageal squamous cell cancer (ESCC)	intravenous PD-1 inhibitor (nivolumab, pembrolizumab, and camrelizumab)
4	Zhigang Wu	2021	China	retrospective study	38	61 (57–75)	Esophageal squamous cell cancer (ESCC)	taxel (docetaxel or paclitaxel) and Platinum Doublet (cisplatin or carboplatin) combined immunotherapy (Camrelizumab, Pembrolizumab, or Sintilimab)
5	Peng Yang	2021	China	prospective study	16	60.5 (56.0-67.3)	Esophageal squamous cell cancer (ESCC)	camrelizumab concurrent with paclitaxel plus carboplatin
6	Zhenyang Zhang	2021	China	open-label, non-randomized, single-arm, single-center phase 2 trial	30	58.3±7.1	Esophageal squamous cell cancer (ESCC)	cisplatin, albumin-bound paclitaxel and sintilimab

7	Hongtao Duan	2022	China	An Open-Label, Single-Arm Study	18	60.9 (35-78)	Esophageal squamous cell carcinoma (ESCC)	pembrolizumab combined with conventional chemotherapy
8	Lei Gao	2022	China	open-label, non-randomized, single-arm, single-center phase II trial	20	58.3 (49–69)	Esophageal squamous cell carcinoma (ESCC)	docetaxel, cisplatin and toripalimab
9	Wenwu He	2021	China	single-arm, open-label, phase II trial	20	62.1 (51.5-72.3)	Esophageal squamous cell carcinoma (ESCC)	toripalimab plus paclitaxel and carboplatin
10	Shujie Huang	2022	China	retrospective study	155	61(55-66)	Esophageal squamous cell carcinoma (ESCC)	PD-1 inhibitors (camrelizumab, pembrolizumab, sintilimab, tislelizumab, toripalimab, and nivolumab) and platinum-based plus docetaxelor taxane-based agents
11	Xiaotao Huang	2022	China	open-label, single-arm, phase Ib clinical trial	23	56 (31–70)	Head and neck squamous cell carcinoma (HNSCC)	toripalimab, gemcitabine and cisplatin
12	Jun Liu	2022	China	multicenter, phase 2 study	56	61 (40-70)	Esophageal squamous cell carcinoma (ESCC)	camrelizumab, nab-paclitaxel and cisplatin
13	Huilai Lv	2023	China	retrospective study	90	65 (49-78)	Esophageal squamous cell carcinoma (ESCC)	sintilimab, albumin-bound paclitaxel and nedaplatin

14	Huilai Lv	2022	China	retrospective study	96	65 (60–69)	Esophageal squamous cell carcinoma (ESCC)	sintilimab with platinum and taxanes
15	Peng Shen	2022	China	retrospective study	30	58 (38–79)	Oral cavity squamous cell carcinoma (OCSCC)	nivolumab plus SBRT
16	Weixiong Yang	2023	China	single-arm, prospective trial	23	58.6±10.1	Esophageal squamous cell carcinoma (ESCC)	camrelizumab, nab-paclitaxel, and carboplatin
17	Yang Yang	2022	China	retrospective cohort study	370	63 (30–81)	Esophageal squamous cell carcinoma (ESCC)	pembrolizumab, camrelizumab, or SHR-1316 with chemotherapy (IO+CT/CRT)
18	Guoqiang Yin	2023	China	retrospective cohort study	34	59 (52–69)	Esophageal squamous cell carcinoma (ESCC)	camrelizumab, paclitaxel and carboplatin
19	Zhanjie Zhang	2023	China	In this single-center, single-arm, phase 2 trial	30	58 (41–69)	Head and neck squamous cell carcinoma (HNSCC)	albumin-bound paclitaxel, cisplatin and camrelizumab
20	Zhi Zhang	2022	China	single-center single-arm, phase II clinical trial	47	66 (64-70)	Esophageal squamous cell carcinoma (ESCC)	sintilimab, paclitaxel liposomes and carboplatin
21	Xiaofeng Chen	2022	China	open-label, single-arm, phase 2 trial	30	64 (42–70)	Esophageal squamous cell carcinoma (ESCC)	sintilimab, liposomal paclitaxel, cisplatin and S-1

22	Yuzhen Chen	2023	China	prospective, single-arm, phase II clinical trial	43	63 (55–67.5)	Esophageal squamous cell carcinoma (ESCC)	sintilimab, cisplatin, carboplatin and paclitaxel liposome or nab-paclitaxel
23	Qi Fang	2023	China	retrospective cohort study	156	60.4	Laryngeal and hypopharyngeal squamous cell carcinoma (SCC)	PD-1 inhibitors and paclitaxel (Albumin-bound) and cisplatin
24	Yingying Huang	2023	China	prospective, single-arm, open-label trial	20	54 (45–63.8)	Oral squamous cell carcinoma (OSCC)	albumin paclitaxel, cisplatin, and toripalimab
25	Shetal A. Patel	2023	USA	phase 2, single-arm, multicenter study	39	60 (38–86)	Head and neck squamous cell carcinoma (HNSCC)	carboplatin, nab-paclitaxel and durvalumab
26	Kai Wang	2023	China	prospective, single-arm, single-centre clinical trial	22	58.1 ± 10.49 (22-69)	Head and neck squamous cell carcinoma (HNSCC)	pembrolizumab combined with cisplatin and paclitaxel
27	Qin Wang	2023	China	retrospective cohort study	38	65 (59-70)	Lung squamous cell carcinoma	neoadjuvant immunotherapy combined with neoadjuvant chemotherapy
28	Pengfei Xu	2023	China	retrospective study	295	57.2 ± 9.5 (20–80)	Oropharyngeal and hypopharyngeal squamous cell	albumin-bound paclitaxel, cisplatin, and a PD-1 inhibitor (Camrelizumab, Sintilimab, Toripalimab,

							carcinoma (OPHSCC)	Pembrolizumab, Nivolumab, or Tislelizumab)
29	Jun Yin	2023	China	phase 1b trial	25	65 (55-75)	Esophageal squamous cell carcinomas (ESCC)	adebrelimab and chemotherapy or chemoradiotherapy
30	Guangyin Zhao	2023	China	retrospective cohort study	16 (SCC subgroup)	65 [42–74]	Lung squamous cell carcinoma subgroup	pembrolizumab, carboplatin or cisplatin, and pemetrexed or nab-paclitaxel
31	Ruiqin Zhou	2023	China	retrospective cohort study	19	65.89 ± 6.06	Esophageal squamous cell carcinoma (ESCC)	camrelizumab, docetaxel and nedaplatin
32	Yiyu Guo	2024	China	retrospective cohort study	132	65 (43–75)	Esophageal squamous cell carcinoma (ESCC)	taxane (docetaxel or paclitaxel) and cisplatin/carboplatin combined with immunotherapy (Sintilimab, Toripalimab, Pembrolizumab Camrelizumab or Tislelizumab)
33	Ning Jiang	2023	China	prospective, single-arm phase Ib study	23	65 (37–72)	Esophageal squamous cell carcinoma (ESCC)	toripalimab combination with paclitaxel and carboplatin
34	Emily Y. Kim	2024	USA	retrospective cohort study	27	72 (53-87)	Cutaneous squamous cell carcinoma (CSCC)	cemiplimab or pembrolizumab and chemotherapy
35	Chunyang Li	2024	China	retrospective observational study	33	64.56 ± 6.805	Esophageal squamous cell carcinoma (ESCC)	paclitaxel, cisplatin/nedaplatin/carboplatin and sintilimab
36	Kezhen Li	2023	China	single-arm, phase 2	85	51 (46–57)	Cervical squamous cell	cisplatin, nab-paclitaxel, intravenously), and

				trial			carcinoma (CSCC)	camrelizumab
37	Can Liu	2024	China	retrospective single-center observational study	122	65 (41–78)	Esophageal squamous cell carcinoma (ESCC)	six PD-(L)1 inhibitors (pembrolizumab, tislelizumab, camrelizumab , sintilimab, nivolumab , and toripalimab), paclitaxel plus platinum-based agents (paclitaxel+ cisplatin/nedaplatin/carboplatin)
38	Huilai Lv	2024	China	retrospective consecutive cohort study	45		Esophageal squamous cell carcinoma (ESCC)	sintilimab combined with chemotherapy (albumin-bound paclitaxel and nedaplatin)
39	Sehhoon Park	2023	Korea	single-center, single-arm, phase II study	20	63 (51-81)	Esophageal squamous cell carcinoma (ESCC)	nivolumab and chemotherapy or chemoradiotherapy
40	Jin Peng	2024	China	retrospective cohort study	71	60.7 (0.97)	Hypopharynx and larynx squamous cell carcinoma (HLSCC)	camrelizumab monoclonal antibody in combination with albumin-bound paclitaxel and cisplatin
41	Ting Qian	2024	China	prospective cohort study	31	65 (49-74)	Esophageal squamous cell carcinoma (ESCC)	toripalimab, paclitaxel, and cisplatin
42	Jianping Wang	2023	China	prospective, single-arm, open-label study	35		Esophageal squamous cell carcinoma (ESCC)	camrelizumab , nab-paclitaxel and carboplatin
43	Xin Xu	2024	China	single-center, single-arm phase Ib study	21	62 (31–74)	Esophageal squamous cell carcinoma (ESCC)	paclitaxel/cisplatin and toripalimab

44	Yingnan Yang	2023	China	prospective, open-label, single-arm, phase 2 trial	75	62 (48, 74)	Esophageal squamous cell carcinoma (ESCC)	camrelizumab, paclitaxel, and nedaplatin
