# Supplementary material for: The Efficacy and Safety of Neoadjuvant Immunotherapy in Pan-Squamous Cell Carcinomas: A Meta-Analysis of Esophageal, Head and Neck, Cervical, Lung, Cutaneous, and Oral Squamous Cell Carcinomas
Source: J Clin Med. 2026 Aug 6;15(15):6113. doi: 10.3390/jcm15156113 (PMC13466374; doi:10.3390/jcm15156113)
Supplement: Supplementary file 1 [file jcm-15-06113-s001.zip › Supplementary Table S6.pdf]

Supplementary Table S6. Methodological quality assessment using the MINORS instrument

Scores: 0, not reported; 1, reported but inadequate; 2, reported and adequate. Non-comparative studies were assessed with eight items (maximum 16), and comparative non-randomized studies with 12 items (maximum 24).

No.	Study	Design	D1	D2	D3	D4	D5	D6	D7	D8	D9	D10	D11	D12	Total	Max.
1	Duan et al., 2021	Multicenter single-arm observational study	2	1	0	1	1	2	2	0	NA	NA	NA	NA	9	16
2	Ferrarotto et al., 2021	Single-institution pilot phase II study	2	1	2	2	1	2	2	2	NA	NA	NA	NA	14	16
3	Shen et al., 2021	Single-center prospective single-group pilot study	2	1	2	1	0	2	2	0	NA	NA	NA	NA	10	16
4	Wu et al., 2021	Single-center retrospective non-comparative cohort	2	1	0	2	1	2	2	0	NA	NA	NA	NA	10	16
5	Yang et al., 2021	Single-center prospective single-arm pilot study	2	1	2	2	0	2	2	0	NA	NA	NA	NA	11	16
6	Zhang et al., 2021 (ESONICT-1)	Investigator-initiated open-label non-randomized single-arm single-center phase II trial	2	2	2	1	0	2	2	0	NA	NA	NA	NA	11	16
7	Duan et al., 2022 (PEN-ICE)	Open-label single-arm study	2	1	1	2	0	2	1	0	NA	NA	NA	NA	9	16
8	Gao et al., 2022 (ESONICT-2)	Investigator-initiated prospective single-center single-arm phase II trial	2	2	2	1	0	2	2	0	NA	NA	NA	NA	11	16
9	He et al., 2021	Prospective single-arm open-label phase II trial	2	1	2	1	1	2	2	0	NA	NA	NA	NA	11	16
10	Huang et al., 2022 (RICE-Retro)	Multicenter real-world retrospective comparative cohort study	2	1	0	2	1	2	1	0	2	0	0	1	12	24
11	Huang et al., 2022 (NeoTGP01)	Open-label prospective single-arm phase Ib clinical trial	2	2	2	2	1	2	2	1	NA	NA	NA	NA	14	16
12	Liu et al., 2022 (NIC-ESCC2019)	Multicenter open-label single-arm phase II study	2	1	2	1	0	2	2	2	NA	NA	NA	NA	12	16
13	Lv et al., 2023	Single-center real-world retrospective survival cohort	2	1	0	2	0	1	0	0	NA	NA	NA	NA	6	16
14	Lv et al., 2022	Single-center retrospective real-world cohort	2	1	0	2	0	1	0	0	NA	NA	NA	NA	6	16
15	Shen et al., 2022	Single-center retrospective non-comparative cohort	2	1	0	2	0	1	2	0	NA	NA	NA	NA	8	16

No.	Study	Design	D1	D2	D3	D4	D5	D6	D7	D8	D9	D10	D11	D12	Total	Max.
16	Yang et al., 2022/2023	Investigator-initiated prospective single-arm pilot trial	2	1	2	2	1	2	1	0	NA	NA	NA	NA	11	16
17	Yang et al., 2022	Multicenter retrospective real-world cohort	2	1	0	2	0	2	2	0	NA	NA	NA	NA	9	16
18	Yin et al., 2023	Single-center retrospective non-comparative cohort	2	1	0	2	0	2	2	0	NA	NA	NA	NA	9	16
19	Zhang Zhanjie et al., 2023	Single-center prospective single-arm phase 2 trial	2	1	2	1	2	2	2	2	NA	NA	NA	NA	14	16
20	Zhang Zhi et al., 2022	Single-center prospective single-arm phase 2 trial	2	1	2	1	1	2	2	2	NA	NA	NA	NA	13	16
21	Chen Xiaofeng et al., 2023	Open-label prospective single-arm phase 2 trial	2	2	2	2	1	2	2	1	NA	NA	NA	NA	14	16
22	Chen Yuzhen et al., 2023	Single-center prospective single-arm phase II trial	2	2	2	1	1	2	2	2	NA	NA	NA	NA	14	16
23	Fang et al., 2023	Single-center retrospective non-comparative cohort	2	2	0	2	0	1	2	0	NA	NA	NA	NA	9	16
24	Huang Yingying et al., 2023	Prospective single-arm open-label trial	2	2	2	2	1	2	2	2	NA	NA	NA	NA	15	16
25	Patel et al., 2023	Multicenter prospective single-arm phase 2 trial	2	1	2	1	2	2	2	2	NA	NA	NA	NA	14	16
26	Wang Kai et al., 2023	Single-center prospective single-arm clinical trial	2	2	2	1	0	2	2	0	NA	NA	NA	NA	11	16
27	Wang Qin et al., 2023	Single-center retrospective comparative cohort	2	1	0	2	1	2	2	0	2	2	1	1	16	24
28	Xu Pengfei et al., 2023	Single-center retrospective comparative cohort	2	1	0	2	0	1	0	0	2	1	1	1	11	24
29	Yin Jun et al., 2023	Prospective single-arm phase 1b trial with post hoc historical comparison	2	1	2	2	2	2	1	2	1	0	2	2	19	24
30	Zhao Guangyin et al., 2023	Single-center retrospective cohort	2	1	0	2	1	2	2	0	NA	NA	NA	NA	10	16
31	Zhou Ruiqin et al., 2023	Single-center retrospective comparative cohort	2	2	0	2	0	2	0	0	2	1	1	1	13	24
32	Guo Yiyu et al., 2024	Single-center real-world retrospective cohort	2	1	0	2	0	2	0	0	NA	NA	NA	NA	7	16

No.	Study	Design	D1	D2	D3	D4	D5	D6	D7	D8	D9	D10	D11	D12	Total	Max.
33	Jiang Ning et al., 2024	Single-center prospective single-arm phase 1b trial	2	2	2	2	2	2	2	1	NA	NA	NA	NA	15	16
34	Kim Emily Y. et al., 2024	Two-center retrospective cohort	2	2	0	1	1	2	2	0	NA	NA	NA	NA	10	16
35	Li Chunyang et al., 2024	Single-center retrospective comparative cohort	2	1	0	2	0	2	0	0	2	2	1	1	13	24
36	Li Kezhen et al., 2024	Multicenter prospective single-arm phase 2 trial	2	2	2	2	2	2	2	2	NA	NA	NA	NA	16	16
37	Liu Can et al., 2024	Single-center real-world retrospective cohort	2	2	0	1	0	1	2	0	NA	NA	NA	NA	8	16
38	Lv Huilai et al., 2024	Single-center retrospective propensity-matched comparative cohort	2	2	0	2	1	2	0	0	2	2	2	2	17	24
39	Park Sehhoon et al., 2024	Single-center prospective single-arm phase 2 trial	2	1	2	1	1	2	2	2	NA	NA	NA	NA	13	16
40	Peng Jin et al., 2024	Single-center retrospective cohort	2	2	0	2	0	1	0	0	NA	NA	NA	NA	7	16
41	Qian Ting et al., 2024	Single-center prospective single-arm study	2	1	2	1	1	2	1	0	NA	NA	NA	NA	10	16
42	Wang Jianping et al., 2024	Prospective single-arm cohort with historical control	2	1	1	2	0	2	2	2	1	0	0	1	14	24
43	Xu Xin et al., 2024	Single-center prospective single-arm phase 1b trial	2	1	2	1	0	2	2	1	NA	NA	NA	NA	11	16
44	Yang Yingnan et al., 2024	Prospective single-arm phase 2 trial	2	1	2	1	0	2	1	2	NA	NA	NA	NA	11	16
