# Supplementary material for: The Efficacy and Safety of Neoadjuvant Immunotherapy in Pan-Squamous Cell Carcinomas: A Meta-Analysis of Esophageal, Head and Neck, Cervical, Lung, Cutaneous, and Oral Squamous Cell Carcinomas
Source: J Clin Med. 2026 Aug 6;15(15):6113. doi: 10.3390/jcm15156113 (PMC13466374; doi:10.3390/jcm15156113)
Supplement: Supplementary file 1 [file jcm-15-06113-s001.zip › Supplementary Table S7.pdf]

Supplementary Table S7. Full database- and registry-specific search strategies

Search period: database inception to October 2024. No language restriction was applied at the search stage. Syntax was adapted to each platform.

Database/platform	Complete strategy
PubMed	((neoadjuvant[Title/Abstract] OR preoperative[Title/Abstract] OR perioperative[Title/Abstract]) AND (immunotherap*[Title/Abstract] OR "immune checkpoint inhibitor*"[Title/Abstract] OR "PD-1"[Title/Abstract] OR "PD-L1"[Title/Abstract] OR pembrolizumab[Title/Abstract] OR nivolumab[Title/Abstract] OR camrelizumab[Title/Abstract] OR sintilimab[Title/Abstract] OR toripalimab[Title/Abstract] OR tislelizumab[Title/Abstract] OR cemiplimab[Title/Abstract] OR durvalumab[Title/Abstract] OR adebrelimab[Title/Abstract]) AND ("Carcinoma, Squamous Cell"[Mesh] OR "squamous cell carcinoma*"[Title/Abstract] OR "squamous cell cancer*"[Title/Abstract]) AND (esophag*[Title/Abstract] OR oesophag*[Title/Abstract] OR "head and neck"[Title/Abstract] OR oral[Title/Abstract] OR oropharyn*[Title/Abstract] OR hypopharyn*[Title/Abstract] OR laryn*[Title/Abstract] OR cervi*[Title/Abstract] OR lung[Title/Abstract] OR pulmonary[Title/Abstract] OR cutaneous[Title/Abstract] OR skin[Title/Abstract]))
Embase (Embase.com)	('neoadjuvant therapy'/exp OR neoadjuvant:ti,ab,kw OR preoperative:ti,ab,kw OR perioperative:ti,ab,kw) AND ('immunotherapy'/exp OR 'immune checkpoint inhibitor'/exp OR immunotherap*:ti,ab,kw OR 'pd 1':ti,ab,kw OR 'pd l1':ti,ab,kw OR pembrolizumab:ti,ab,kw OR nivolumab:ti,ab,kw OR camrelizumab:ti,ab,kw OR sintilimab:ti,ab,kw OR toripalimab:ti,ab,kw OR tislelizumab:ti,ab,kw OR cemiplimab:ti,ab,kw OR durvalumab:ti,ab,kw OR adebrelimab:ti,ab,kw) AND ('squamous cell carcinoma'/exp OR 'squamous cell carcinoma*':ti,ab,kw OR 'squamous cell cancer*':ti,ab,kw) AND (esophag*:ti,ab,kw OR oesophag*:ti,ab,kw OR 'head and neck':ti,ab,kw OR oral:ti,ab,kw OR oropharyn*:ti,ab,kw OR hypopharyn*:ti,ab,kw OR laryn*:ti,ab,kw OR cervi*:ti,ab,kw OR lung:ti,ab,kw OR pulmonary:ti,ab,kw OR cutaneous:ti,ab,kw OR skin:ti,ab,kw)
Cochrane Library	([mh "Carcinoma, Squamous Cell"] OR "squamous cell carcinoma*":ti,ab,kw OR "squamous cell cancer*":ti,ab,kw) AND (neoadjuvant:ti,ab,kw OR preoperative:ti,ab,kw OR perioperative:ti,ab,kw) AND (immunotherap*:ti,ab,kw OR "immune checkpoint inhibitor*":ti,ab,kw OR "PD-1":ti,ab,kw OR "PD-L1":ti,ab,kw OR pembrolizumab:ti,ab,kw OR nivolumab:ti,ab,kw OR camrelizumab:ti,ab,kw OR sintilimab:ti,ab,kw OR toripalimab:ti,ab,kw OR tislelizumab:ti,ab,kw OR cemiplimab:ti,ab,kw OR durvalumab:ti,ab,kw OR adebrelimab:ti,ab,kw)
ClinicalTrials.gov	AREA[ConditionSearch] ("squamous cell carcinoma" OR "squamous cell cancer") AND AREA[InterventionSearch] (immunotherapy OR "immune checkpoint inhibitor" OR PD-1 OR PD-L1 OR pembrolizumab OR nivolumab OR camrelizumab OR sintilimab OR toripalimab OR tislelizumab OR cemiplimab OR durvalumab OR adebrelimab) AND AREA[OtherTerms] (neoadjuvant OR preoperative OR perioperative)
WHO ICTRP	Condition: "squamous cell carcinoma" OR "squamous cell cancer"; Intervention: immunotherapy OR "immune checkpoint inhibitor" OR PD-1 OR PD-L1 OR pembrolizumab OR nivolumab OR camrelizumab OR sintilimab OR toripalimab OR tislelizumab OR cemiplimab OR durvalumab OR adebrelimab; Recruitment title/keyword: neoadjuvant OR preoperative OR perioperative
Other sources	Backward reference-list screening of all included reports and relevant systematic reviews; overlapping reports were reconciled at the cohort and outcome level.
