# Supplementary material for: The Efficacy and Safety of Neoadjuvant Immunotherapy in Pan-Squamous Cell Carcinomas: A Meta-Analysis of Esophageal, Head and Neck, Cervical, Lung, Cutaneous, and Oral Squamous Cell Carcinomas
Source: J Clin Med. 2026 Aug 6;15(15):6113. doi: 10.3390/jcm15156113 (PMC13466374; doi:10.3390/jcm15156113)
Supplement: Supplementary file 1 [file jcm-15-06113-s001.zip › Supplementary Tables S2-S5.pdf]

Supplementary Tables

Supplementary Table S2. Study-by-outcome matrix.

No.	Study	Cancer site	Study design	Sample size	ORR	cCR	pCR	PR	MPR	1-y PFS	1-y DFS	1-y OS	Grade 3–4 AE	Notes
1	Hongtao Duan 2021	Lung squamous cell carcinoma subgroup	single-arm observational study	19 (SCC subgroup)	Yes	No	Yes	Yes	Yes	No	No	No	No	Only the separately extractable SCC subgroup was used.
2	Renata Ferrarotto 2021	Head and neck squamous cell carcinoma (HNSCC)	single-institution, pilot phase II study	20	Yes	No	Yes	No	Yes	No	Yes	Yes	Yes	
3	Dijian Shen 2021	Esophageal squamous cell cancer (ESCC)	single-group study	28	No	Yes	Yes	No	No	No	No	No	Yes	
4	Zhigang Wu 2021	Esophageal squamous cell cancer (ESCC)	retrospective study	38	No	No	Yes	No	Yes	No	No	No	No	
5	Peng Yang 2021	Esophageal squamous cell cancer (ESCC)	prospective study	16	Yes	Yes	Yes	Yes	No	Yes	No	Yes	No	
6	Zhenyang Zhang 2021	Esophageal squamous cell cancer (ESCC)	open-label, non-randomized, single-arm, single-center phase 2 trial	30	Yes	No	Yes	No	Yes	No	No	No	Yes	
7	Hongtao Duan 2022	Esophageal squamous cell carcinoma (ESCC)	An Open-Label, Single-Arm Study	18	No	No	Yes	No	Yes	No	No	No	Yes	
8	Lei Gao 2022	Esophageal squamous cell carcinoma (ESCC)	open-label, non-randomized, single-arm, single-center phase II trial	20	Yes	No	No	No	No	No	No	No	No	
9	Wenwu He 2021	Esophageal squamous cell	single-arm, open-label,	20	No	No	Yes	No	Yes	No	No	No	Yes	

		carcinoma (ESCC)	phase II trial									
10	Shujie Huang 2022	Esophageal squamous cell carcinoma (ESCC)	retrospective study	155	No	No	Yes	No	Yes	No	No	No	Yes
11	Xiaotao Huang 2022	Head and neck squamous cell carcinoma (HNSCC)	open-label, single-arm, phase Ib clinical trial	23	Yes	Yes	No	Yes	No	No	No	No	No
12	Jun Liu 2022	Esophageal squamous cell carcinoma (ESCC)	multicenter, phase 2 study	56	No	No	No	Yes	No	No	No	No	Yes
13	Huilai Lv 2023	Esophageal squamous cell carcinoma (ESCC)	retrospective study	90	No	No	No	No	Yes	No	No	No	Yes
14	Huilai Lv 2022	Esophageal squamous cell carcinoma (ESCC)	retrospective study	96	No	No	Yes	No	No	No	Yes	No	Yes
15	Peng Shen 2022	Oral cavity squamous cell carcinoma (OCSCC)	retrospective study	30	No	Yes	Yes	Yes	Yes	No	No	No	Yes
16	Weixiong Yang, 2023	Esophageal squamous cell carcinoma (ESCC)	single-arm, prospective trial	23	Yes	Yes	Yes	Yes	Yes	No	No	No	Yes
17	Yang Yang 2022	Esophageal squamous cell carcinoma (ESCC)	retrospective cohort study	370	No	No	Yes	No	No	No	No	No	Yes
18	Guoqiang Yin 2023	Esophageal squamous cell carcinoma (ESCC)	retrospective cohort study	34	No	Yes	Yes	Yes	Yes	No	Yes	Yes	No
19	Zhanjie Zhang 2023	Head and neck squamous cell carcinoma (HNSCC)	In this single-center, single-arm, phase 2 trial	30	No	No	Yes	No	Yes	No	Yes	No	Yes

20	Zhi Zhang 2022	Esophageal squamous cell carcinoma (ESCC)	single-center single-arm, phase II clinical trial	47	No	No	Yes	No	Yes	No	Yes	Yes	No	
21	Xiaofeng Chen 2022	Esophageal squamous cell carcinoma (ESCC)	open-label, single-arm, phase 2 trial	30	No	No	Yes	No	Yes	No	No	No	Yes	
22	Yuzhen Chen 2023	Esophageal squamous cell carcinoma (ESCC)	prospective, single-arm, phase II clinical trial	43	No	Yes	Yes	Yes	Yes	No	No	No	No	
23	Qi Fang 2023	Laryngeal and hypopharyngeal squamous cell carcinoma (SCC)	retrospective cohort study	156	No	No	No	No	No	Yes	No	Yes	No	
24	Yingying Huang 2023	Oral squamous cell carcinoma (OSCC)	prospective, single-arm, open-label trial	20	No	Yes	Yes	Yes	Yes	No	No	No	Yes	
25	Shetal A. Patel 2023	Head and neck squamous cell cancer(HNSCC)	phase 2, single-arm, multicenter study	39	Yes	Yes	No	Yes	No	Yes	No	Yes	No	
26	Kai Wang 2023	Head and neck squamous cell carcinoma (HNSCC)	prospective, single-arm, single-centre clinical trial	22	No	No	No	No	No	No	No	No	Yes	
27	Qin Wang 2023	Lung squamous cell carcinoma	retrospective cohort study	38	No	No	No	No	No	No	No	No	No	
28	Pengfei Xu 2023	Oropharyngeal and hypopharyngeal squamous cell carcinoma (OPHSCC)	retrospective study	295	Yes	No	No	Yes	No	Yes	No	Yes	No	
29	Jun Yin 2023	Esophageal squamous cell carcinomas (ESCC)	phase 1b trial	25	No	No	Yes	No	Yes	No	No	No	No	
30	Guangyin	Lung squamous	retrospective	16 (SCC	No	No	No	No	No	No	No	No	No	Characteristics

	Zhao 2023	cell carcinoma subgroup	cohort study	subgroup)										only; SCC-specific endpoint events were not separately extractable.
31	Ruiqin Zhou 2023	Esophageal squamous cell carcinoma (ESCC)	retrospective cohort study	19	No	No	Yes	No	No	No	No	No	Yes	
32	Yiyu Guo 2024	Esophageal squamous cell carcinoma (ESCC)	retrospective cohort study	132	Yes	No	Yes	No	No	No	Yes	Yes	Yes	
33	Ning Jiang 2023	Esophageal squamous cell carcinoma (ESCC)	prospective, single-arm phase Ib study	23	No	No	Yes	No	Yes	No	No	No	No	
34	Emily Y. Kim, BS 2024	Cutaneous squamous cell carcinoma (CSCC)	retrospective cohort study	27	Yes	No	Yes	No	No	Yes	No	Yes	No	
35	Chunyang Li 2024	Esophageal squamous cell carcinoma (ESCC)	retrospective observational study	33	No	Yes	Yes	No	No	No	No	No	No	
36	Kezhen Li 2023	Cervical cancer	single-arm, phase 2 trial	85	Yes	No	Yes	Yes	No	No	No	No	No	
37	Can Liu 2024	Esophageal squamous cell carcinoma (ESCC)	retrospective single-center observational study	122	No	No	Yes	No	Yes	No	Yes	Yes	Yes	
38	Huilai Lv 2024	Esophageal squamous cell carcinoma (ESCC)	retrospective consecutive cohort study	45	No	No	Yes	No	Yes	No	No	Yes	Yes	
39	Sehhoon Park 2023	Esophageal squamous cell carcinoma (ESCC)	single-center, single-arm, phase II study	20	No	No	No	Yes	Yes	No	No	No	No	
40	Jin Peng 2024	Hypopharynx and larynx	retrospective cohort study	71	Yes	No	No	No	No	Yes	No	Yes	No	

		squamous cell carcinoma (HLSCC)											
41	Ting Qian 2024	Esophageal squamous cell carcinoma (ESCC)	prospective cohort study	31	No	No	Yes	Yes	No	Yes	No	Yes	No
42	Jianping Wang 2023	Esophageal squamous cell carcinoma (ESCC)	prospective, single-arm, open-label study	35	No	No	Yes	No	Yes	No	Yes	Yes	No
43	Xin Xu 2024	Esophageal squamous cell carcinoma (ESCC)	single-center, single-arm phase Ib study	21	No	No	Yes	No	Yes	No	No	No	No
44	Yingnan Yang 2023	Esophageal squamous cell carcinoma (ESCC)	prospective, open-label, single-arm, phase 2 trial	75	Yes	Yes	Yes	No	Yes	No	No	No	No

Supplementary Table S3. Leave-one-out sensitivity analyses.

Outcome	Omitted study	Pooled	Lower 95% CI	Upper 95% CI	I^2 (%)
pCR	Hongtao Duan 2021 (SCC subgroup)	0.303	0.267	0.342	39.1
pCR	Ferrarotto 2021	0.295	0.260	0.332	33.9
pCR	Shen 2021	0.300	0.264	0.340	39.8
pCR	Wu 2021	0.300	0.263	0.339	39.5
pCR	Yang 2021	0.301	0.264	0.340	40.0
pCR	Zhang 2021	0.304	0.267	0.343	38.7
pCR	Duan 2022	0.299	0.263	0.337	38.5
pCR	Gao 2020	0.302	0.266	0.341	39.4
pCR	He 2021	0.303	0.266	0.342	39.4
pCR	Huang 2022	0.303	0.267	0.342	39.0
pCR	Lv 2022	0.302	0.264	0.342	39.9
pCR	Shen 2022	0.290	0.258	0.324	24.5
pCR	W. Yang 2023	0.302	0.265	0.341	39.9
pCR	Y. Yang 2022	0.305	0.266	0.347	38.3
pCR	Yin 2023	0.299	0.263	0.339	39.4
pCR	Z. Zhang 2023	0.299	0.263	0.338	39.2
pCR	Zhi Zhang 2022	0.304	0.267	0.344	39.0
pCR	X. Chen 2022	0.304	0.267	0.343	38.9
pCR	Y. Chen 2023	0.302	0.265	0.342	40.0
pCR	Y. Huang 2023	0.301	0.265	0.341	40.0
pCR	Wang 2023	0.300	0.263	0.339	39.5
pCR	Jun Yin 2023	0.303	0.268	0.341	36.5
pCR	Zhou 2023	0.302	0.265	0.341	39.9
pCR	Guo 2024	0.305	0.268	0.346	38.2
pCR	Jiang 2023	0.295	0.260	0.332	33.9
pCR	Kim 2024	0.297	0.262	0.335	37.3
pCR	Li 2024	0.300	0.263	0.339	39.5
pCR	Kezhen Li 2023	0.307	0.270	0.346	36.0
pCR	Liu 2024	0.304	0.266	0.345	39.6
pCR	Lv 2024	0.307	0.270	0.346	36.0
pCR	Qian 2024	0.302	0.265	0.341	40.0
pCR	J. Wang 2023	0.293	0.259	0.329	31.5
pCR	Xu 2024	0.297	0.262	0.335	37.3
pCR	Y. Yang 2023	0.303	0.265	0.343	39.9
MPR	Hongtao Duan 2021 (SCC subgroup)	0.471	0.409	0.535	57.4
MPR	Ferrarotto 2021	0.478	0.419	0.538	53.8
MPR	Wu 2021	0.473	0.410	0.537	57.2

MPR	Zhang 2021	0.468	0.406	0.531	57.3
MPR	Duan 2022	0.465	0.404	0.527	56.1
MPR	Gao 2020	0.471	0.410	0.534	57.4
MPR	He 2021	0.471	0.409	0.534	57.4
MPR	Huang 2022	0.476	0.415	0.538	56.0
MPR	Lv 2023	0.459	0.400	0.518	47.8
MPR	Shen 2022	0.476	0.415	0.539	56.1
MPR	W. Yang 2023	0.469	0.407	0.532	57.4
MPR	Yin 2023	0.469	0.406	0.533	57.4
MPR	Z. Zhang 2023	0.460	0.401	0.520	52.5
MPR	Zhi Zhang 2022	0.472	0.408	0.536	57.4
MPR	X. Chen 2022	0.469	0.406	0.532	57.4
MPR	Y. Chen 2023	0.474	0.412	0.538	56.8
MPR	Y. Huang 2023	0.466	0.404	0.528	56.6
MPR	Wang 2023	0.467	0.405	0.530	57.1
MPR	Jun Yin 2023	0.479	0.418	0.540	54.5
MPR	Jiang 2023	0.461	0.402	0.520	52.7
MPR	Liu 2024	0.479	0.417	0.541	52.6
MPR	Lv 2024	0.475	0.412	0.540	55.8
MPR	Park 2023	0.477	0.418	0.538	54.6
MPR	J. Wang 2023	0.457	0.400	0.515	49.5
MPR	Xu 2024	0.470	0.408	0.533	57.5
MPR	Y. Yang 2023	0.471	0.407	0.536	57.4
ORR	Hongtao Duan 2021 (SCC subgroup)	0.696	0.573	0.796	83.1
ORR	Renata Ferrarotto 2021	0.727	0.615	0.816	81.2
ORR	Peng Yang 2021	0.696	0.575	0.795	83.1
ORR	Zhenyang Zhang 2021	0.706	0.582	0.806	83.4
ORR	Xiaotao Huang 2022	0.720	0.602	0.813	82.5
ORR	Weixiong Yang 2023	0.690	0.570	0.789	82.8
ORR	Lei Gao 2022	0.704	0.581	0.803	83.3
ORR	Pengfei Xu 2023	0.690	0.566	0.791	79.1
ORR	Yiyu Guo 2024	0.716	0.584	0.819	82.3
ORR	Emily Y. Kim 2024	0.716	0.596	0.811	83.0
ORR	Kezhen Li 2023	0.663	0.553	0.757	77.5
ORR	Jin Peng 2024	0.677	0.559	0.776	80.0
ORR	Yingnan Yang 2023 (cohort 1)	0.721	0.602	0.816	80.8
ORR	Shetal A. Patel 2023	0.715	0.591	0.813	83.0
cCR	Dijian Shen 2021	0.151	0.086	0.253	64.2
cCR	Peng Yang 2021	0.165	0.091	0.279	70.6
cCR	Xiaotao Huang 2022	0.189	0.111	0.302	66.8
cCR	Peng Shen 2022	0.181	0.104	0.298	68.7

cCR	Weixiong Yang 2023	0.190	0.112	0.303	66.5
cCR	Guoqiang Yin 2023	0.189	0.111	0.303	66.5
cCR	Yuzhen Chen 2023	0.187	0.108	0.304	65.8
cCR	Yingying Huang 2023	0.179	0.103	0.293	69.6
cCR	Chunyang Li 2024	0.160	0.086	0.279	70.5
cCR	Yingnan Yang 2023	0.152	0.090	0.246	55.9
cCR	Shetal A. Patel 2023	0.168	0.091	0.289	70.3
PR	Hongtao Duan 2021 (SCC subgroup)	0.573	0.479	0.662	68.4
PR	Peng Yang 2021	0.589	0.492	0.679	70.4
PR	Xiaotao Huang 2022	0.600	0.506	0.687	68.3
PR	Jun Liu 2022	0.593	0.493	0.686	69.6
PR	Peng Shen 2022	0.597	0.500	0.686	69.0
PR	Weixiong Yang 2023	0.572	0.480	0.659	67.6
PR	Guoqiang Yin 2023	0.590	0.491	0.682	70.2
PR	Yuzhen Chen 2023	0.596	0.498	0.687	68.8
PR	Yingying Huang 2023	0.593	0.497	0.683	69.9
PR	Shetal A. Patel 2023	0.605	0.514	0.690	64.8
PR	Pengfei Xu 2023	0.579	0.474	0.678	68.7
PR	Kezhen Li 2023	0.562	0.475	0.646	58.6
PR	Sehhoon Park 2023	0.603	0.513	0.687	66.6
PR	Ting Qian 2024	0.570	0.477	0.659	67.3
AE	Renata Ferrarotto 2021	0.186	0.144	0.235	68.2
AE	Dijian Shen 2021	0.186	0.145	0.237	68.1
AE	Zhenyang Zhang 2021	0.187	0.146	0.237	67.3
AE	Hongtao Duan 2022	0.177	0.136	0.227	69.1
AE	Wenwu He 2021	0.179	0.138	0.230	69.4
AE	Shujie Huang 2022	0.173	0.133	0.223	64.1
AE	Jun Liu 2022	0.187	0.145	0.238	67.6
AE	Huilai Lv 2023	0.175	0.133	0.226	68.5
AE	Huilai Lv 2022	0.186	0.143	0.238	67.4
AE	Peng Shen 2022	0.181	0.139	0.233	69.3
AE	Weixiong Yang, 2023	0.172	0.134	0.217	63.4
AE	Yang Yang 2022	0.188	0.145	0.239	62.4
AE	Zhanjie Zhang 2023	0.187	0.146	0.237	67.3
AE	Xiaofeng Chen 2022	0.173	0.134	0.221	66.7
AE	Yingying Huang 2023	0.182	0.140	0.233	69.3
AE	Kai Wang 2023	0.185	0.143	0.235	68.6
AE	Ruiqin Zhou 2023	0.185	0.144	0.235	68.3
AE	Yiyu Guo 2024	0.175	0.132	0.227	68.7
AE	Can Liu 2024	0.177	0.134	0.230	69.4
AE	Huilai Lv 2024	0.184	0.142	0.236	68.7

PFS	Peng Yang 2021	0.828	0.784	0.865	24.2
PFS	Qi Fang 2023	0.839	0.787	0.880	18.1
PFS	Shetal A. Patel 2023	0.828	0.782	0.866	24.1
PFS	Pengfei Xu 2023	0.841	0.786	0.883	15.4
PFS	Emily Y. Kim, BS 2024	0.827	0.783	0.863	23.5
PFS	Jin Peng 2024	0.811	0.776	0.841	0.0
PFS	Ting Qian 2024	0.822	0.780	0.858	17.2
DFS	Renata Ferrarotto 2021	0.853	0.730	0.925	86.3
DFS	Huilai Lv 2022	0.833	0.714	0.908	81.3
DFS	Guoqiang Yin 2023	0.860	0.734	0.931	86.5
DFS	Zhanjie Zhang 2023	0.842	0.721	0.916	85.4
DFS	Zhi Zhang 2022	0.879	0.758	0.944	85.8
DFS	Yiyu Guo 2024	0.880	0.769	0.941	80.1
DFS	Can Liu 2024	0.831	0.716	0.906	79.1
DFS	Jianping Wang 2023	0.876	0.755	0.942	86.4
OS	Renata Ferrarotto 2021	0.916	0.897	0.932	0.0
OS	Peng Yang 2021	0.916	0.897	0.932	0.0
OS	Guoqiang Yin 2023	0.916	0.897	0.932	0.0
OS	Zhi Zhang 2022	0.917	0.898	0.933	0.0
OS	Qi Fang 2023	0.913	0.892	0.930	0.0
OS	Shetal A. Patel 2023	0.916	0.897	0.932	0.0
OS	Pengfei Xu 2023	0.914	0.891	0.933	0.0
OS	Yiyu Guo 2024	0.920	0.901	0.936	0.0
OS	Emily Y. Kim, BS 2024	0.918	0.900	0.934	0.0
OS	Can Liu 2024	0.917	0.897	0.934	0.0
OS	Huilai Lv 2024	0.919	0.900	0.935	0.0
OS	Jin Peng 2024	0.917	0.897	0.933	0.0
OS	Ting Qian 2024	0.915	0.896	0.931	0.0
OS	Jianping Wang 2023	0.916	0.897	0.932	0.0

Supplementary Table S4. Exploratory anatomical-site subgroup analyses.

Outcome	Factor	Subgroup	k	Pooled	Lower 95% CI	Upper 95% CI	I² (%)	P between
pCR	Anatomical site	Esophageal	25	0.284	0.252	0.318	17.4	0.274
pCR	Anatomical site	Non-esophageal	9	0.349	0.242	0.474	62.4	0.274
pCR	Checkpoint target	PD-1	32	0.308	0.270	0.350	36.5	0.073
pCR	Checkpoint target	PD-L1	1	0.080	0.015	0.339	0	0.073
MPR	Anatomical site	Esophageal	19	0.474	0.408	0.541	56.1	0.770
MPR	Anatomical site	Non-esophageal	7	0.449	0.303	0.604	61.0	0.770
MPR	Checkpoint target	PD-1	25	0.479	0.418	0.540	54.5	0.054
MPR	Checkpoint target	PD-L1	1	0.240	0.099	0.476	0	0.054
ORR	Anatomical site	Esophageal	6	0.634	0.521	0.733	48.5	0.386
ORR	Anatomical site	Non-esophageal	8	0.725	0.534	0.858	86.4	0.386
ORR	Checkpoint target	PD-1	13	0.715	0.591	0.813	83.0	0.158
ORR	Checkpoint target	PD-L1	1	0.568	0.393	0.727	0	0.158
cCR	Anatomical site	Esophageal	7	0.209	0.107	0.366	73.5	0.281
cCR	Anatomical site	Non-esophageal	4	0.130	0.071	0.226	0	0.281
cCR	Checkpoint target	PD-1	10	0.168	0.091	0.289	70.3	0.806
cCR	Checkpoint target	PD-L1	1	0.189	0.085	0.369	0	0.806
PR	Anatomical site	Esophageal	7	0.583	0.454	0.702	57.2	0.960
PR	Anatomical site	Non-esophageal	7	0.588	0.452	0.712	75.8	0.960
PR	Checkpoint target	PD-1	13	0.605	0.514	0.690	64.8	0.029
PR	Checkpoint target	PD-L1	1	0.380	0.227	0.561	0	0.029
AE	Anatomical site	Esophageal	15	0.199	0.152	0.256	72.2	0.063
AE	Anatomical site	Non-esophageal	5	0.115	0.066	0.192	0	0.063
AE	Checkpoint target	PD-1	19	0.188	0.145	0.239	62.4	
PFS	Anatomical site	Esophageal	2	0.849	0.715	0.926	0	0.714
PFS	Anatomical site	Non-esophageal	5	0.827	0.777	0.868	33.7	0.714
PFS	Checkpoint target	PD-1	6	0.828	0.782	0.866	24.1	0.993
PFS	Checkpoint target	PD-L1	1	0.829	0.667	0.921	0	0.993
DFS	Anatomical site	Esophageal	6	0.834	0.699	0.915	87.3	0.166
DFS	Anatomical site	Non-esophageal	2	0.931	0.807	0.978	0	0.166
DFS	Checkpoint target	PD-1	8	0.857	0.747	0.925	84.5	
OS	Anatomical site	Esophageal	8	0.907	0.876	0.931	0	0.321
OS	Anatomical site	Non-esophageal	6	0.924	0.900	0.943	0	0.321
OS	Checkpoint target	PD-1	13	0.916	0.897	0.932	0	0.571
OS	Checkpoint target	PD-L1	1	0.943	0.798	0.986	0	0.571

Supplementary Table S5. Exploratory Egger regression tests for small-study effects.

Outcome	k	Egger intercept	P value
pCR	34	0.530	0.000
MPR	26	0.110	0.591
ORR	14	1.126	0.543
cCR	11	-4.222	0.075
PR	14	-0.873	0.149
AE	20	-1.317	0.001
OS	14	0.443	0.000
